# The Modulation of Corticospinal Excitability during Motor Imagery of Actions with Objects

**DOI:** 10.1371/journal.pone.0026006

**Published:** 2011-10-13

**Authors:** Nobuaki Mizuguchi, Masanori Sakamoto, Tetsuro Muraoka, Kento Nakagawa, Shoichi Kanazawa, Hiroki Nakata, Noriyoshi Moriyama, Kazuyuki Kanosue

**Affiliations:** 1 Graduate School of Sport Sciences, Waseda University, Mikajima, Tokorozawa, Saitama, Japan; 2 Research Fellow of the Japan Society for the Promotion of Science, Chiyoda-ku, Tokyo, Japan; 3 Department of Physical Education, Faculty of Education, Kumamoto University, Kumamoto, Japan; 4 College of Economics, Nihon University, Chiyoda-Ku, Tokyo, Japan; 5 Faculty of Sport Sciences, Waseda University, Mikajima, Tokorozawa, Saitama, Japan; French National Centre for Scientific Research, France

## Abstract

We investigated whether corticospinal excitability during motor imagery of actions (the power or the pincer grip) with objects was influenced by actually touching objects (tactile input) and by the congruency of posture with the imagined action (proprioceptive input). Corticospinal excitability was assessed by monitoring motor evoked potentials (MEPs) in the first dorsal interosseous following transcranial magnetic stimulation over the motor cortex. MEPs were recorded during imagery of the power grip of a larger-sized ball (7 cm) or the pincer grip of a smaller-sized ball (3 cm)^—^with or without passively holding the larger-sized ball with the holding posture or the smaller-sized ball with the pinching posture. During imagery of the power grip, MEPs amplitude was increased only while the actual posture was the same as the imagined action (the holding posture). On the other hand, during imagery of the pincer grip while touching the ball, MEPs amplitude was enhanced in both postures. To examine the pure effect of touching (tactile input), we recorded MEPs during imagery of the power and pincer grip while touching various areas of an open palm with a flat foam pad. The MEPs amplitude was not affected by the palmer touching. These findings suggest that corticospinal excitability during imagery with an object is modulated by actually touching an object through the combination of tactile and proprioceptive inputs.

## Introduction

Motor imagery is defined as the mental execution of an action without any overt movement or muscle activation. Motor imagery appears to improve motor performance during skill acquisition in sports or in the recovery of motor function following a stroke [1], [2]. During motor imagery, corticospinal excitability is increased, as estimated from the amplitude of motor evoked potentials (MEPs) in response to transcranial magnetic stimulation (TMS) [3]–[8].

Visual and somatosensory information influences brain activity during motor imagery [9]–[14]. The MEPs amplitude during imagery of a finger opposition task with the same posture as the imagined action was greater than that with a different posture [12]. The authors suggested that proprioceptive information affected the process of motor imagery. We recently demonstrated that corticospinal excitability during imagery of an action with an object (power grip on a foam ball) was enhanced by passively holding the object with the same posture as the imagined action [7]. This suggested that motor imagery utilizing an object might have been influenced by tactile input. However, it is unclear whether corticospinal excitability during imagery of actions with objects was modulated solely by tactile input generated by touching the object or by a combination of the tactile input and proprioceptive input that accompanied the posture of holding the object.

The aim of the present study was to examine the influence of tactile and proprioceptive inputs on corticospinal excitability during imagery of actions with objects. As an experimental model, we utilized two actions. The first one was a “power grip” of a larger sized ball (7 cm diameter), the same action as used in a previous study [7]. The second action involved a “pincer grip” of a smaller sized ball (3 cm diameter). These two types of grips have been compared from many different viewpoints [15]–[18]. For example, MEPs amplitude in the first dorsal interroseous (FDI) during a pincer (precision) grip was larger than that which occurred during a power grip under the same background EMG level. This suggested that excitability of the corticospinal tract for the FDI was different between the pincer and power grips [17]. In the present study, subjects were asked to imagine the “power grip” of a larger sized ball (experiment 1) or the “pincer grip” of a smaller sized ball (experiment 2) while holding the larger sized ball in a “holding posture” or the smaller sized ball in a “pinching posture”. To test the influence of the postures themselves or just passively holding the ball, we examined corticospinal excitability during the same conditions as the experiment 1 and 2 but without motor imagery (experiment 3). Finally, to examine the unique contribution of tactile signals, we investigated corticospinal excitability during imagery of the power grip of the larger sized ball (experiment 4) and the pincer grip of the smaller sized ball (experiment 5) while various areas of an open palm were in contact with a flat foam pad.

## Materials and Methods

### Subjects

Twelve healthy volunteers (21–25 years old) participated in both Experiments 1 and 2. Ten healthy volunteers (21–28 years old) participated in Experiment 3. Twelve healthy volunteers (22–31 year old) participated in experiment 4 and twelve healthy volunteers (21–31 years old) participated in Experiment 5. Written informed consent was obtained from all subjects. The study was approved by the Human Research Ethics committee of the Faculty of Sport Sciences, Waseda University. The experiments were conducted in accordance with the Declaration of Helsinki.

### Transcranial magnetic stimulation

TMS was delivered using a Magnetic Stimulator (SMN-1200, Nihon kohden, Japan) connected to a 140 mm round coil of 0.67 T peak magnetic field. In order to stimulate the hand and forearm areas of the left primary motor cortex the center of the coil was placed close to the vertex. The coil was placed at a site determined to be optimal for evoking MEPs in the right first dorsal interosseous muscle (FDI). This site was determined by relocating the coil until the largest MEP was obtained. The current flow in TMS was clockwise when viewed from the top. The resting motor threshold was defined as the lowest TMS intensity that elicited five MEPs in the FDI greater than 50 µV in a series of 10 stimuli. The test TMS intensity was set at 120% of the resting motor threshold. The peak-to-peak amplitudes of the background electromyographic (EMG) signal in a 50 ms window were measured just before the TMS was delivered. Trials with background EMG activity greater than 20 µV were eliminated from the analysis.

### Electromyography

MEPs following single-pulse TMS of the left primary motor cortex were simultaneously recorded from four right hand and forearm muscles (first dorsal interosseous muscle: FDI, abductor digiti minimi muscle: ADM, extensor carpi radialis muscle: ECR, and flexor carpi radialis muscle: FCR). Two Ag-AgCl surface electrodes (1 cm diameter) were positioned on the muscle belly. For the FDI, an electrode was positioned on the muscle belly and another on the metacarpophalangeal joint.

The EMG responses were amplified using an amplifier (MEB-2216, Nihon kohden, Japan) and filtered with a band pass filter of 5–1500 Hz. All signals were converted into a digital format with an A/D converter system (Power lab, ADInstruments, Japan) at 4000 Hz for later analysis.

### Two postures utilized

**Figure 1:**
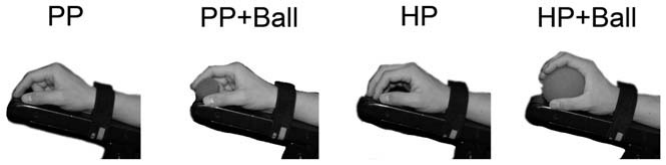
Four types of holding conditions. Pinching Posture (PP), Pinching Posture with a smaller ball (PP+Ball), Holding Posture (HP) and Holding Posture with a larger ball (HP+Ball).

**Figure 2:**
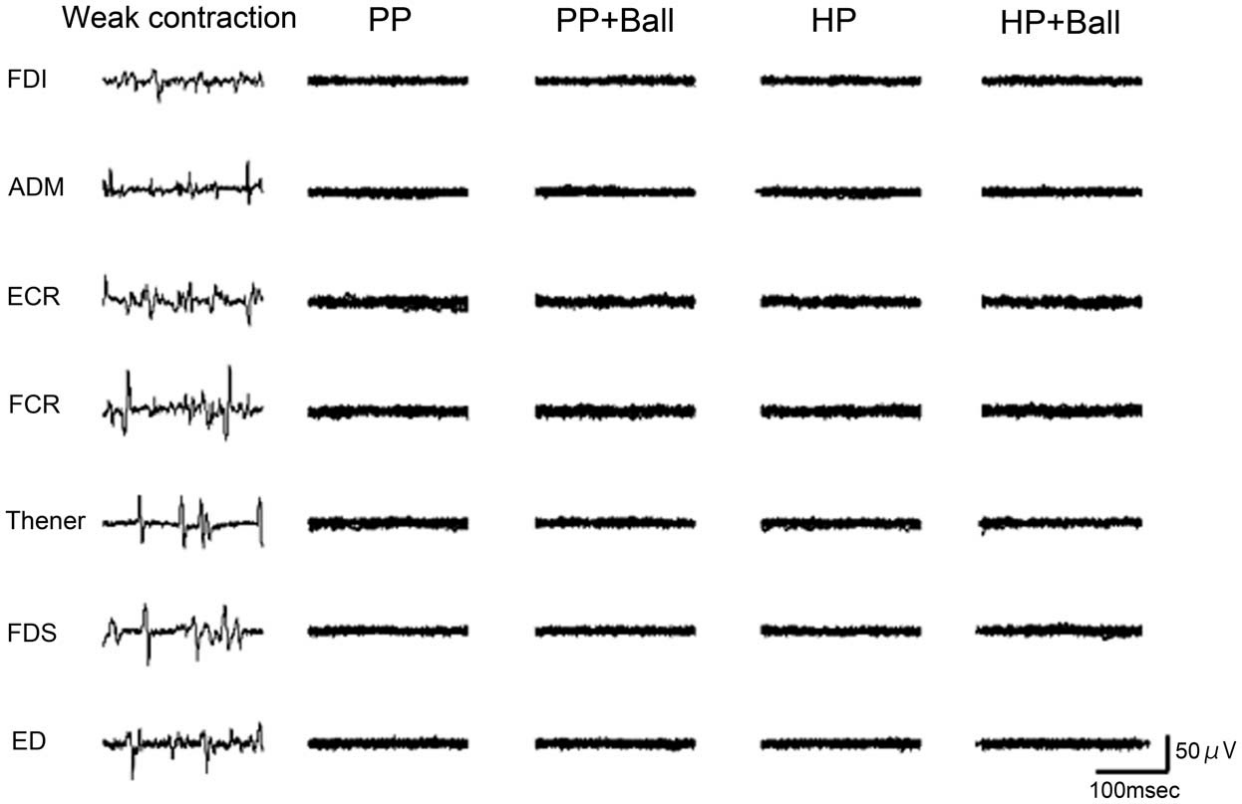
EMG activities during the holding conditions. Superposition of ten resting EMG in seven muscles (first dorsal interosseous muscle: FDI, abductor digiti minimi muscle: ADM, extensor carpi radialis muscle: ECR, and flexor carpi radialis muscle: FCR, Thenar, Flexor digitorum superficialis: FDS and Extensor digitorum: ED muscles) in four conditions; Pinching Posture (PP), Pinching Posture with a smaller ball (PP+Ball), Holding Posture (HP) and Holding Posture with a larger ball (HP+Ball). The leftmost records are the EMG activity during the weakest contraction of each muscle, not necessarily in the same task.

In order to comprehend the rationale and methodology of the experiments, it is critical to understand the two hand postures utilized. Figure 1 illustrates what we will term the “pinching posture (PP)” and the “holding posture (HP)” with or without passively holding a ball. In all conditions, the arm and fingers were put on the armrest to ensure muscle inactiveness. The smaller sized foam ball could be passively maintained in position by the pinching postures without active muscle contraction, because the distance between the thumb and index finger is slightly smaller than the diameter (3 cm) of the ball. Likewise, in the holding posture the roughly spherical shape formed by the inner surface of the hand is slightly smaller than the shape of the outer spherical surface of the larger sized foam ball (7 cm diameter). In a preliminary experiment, we recorded EMG activities in the four conditions at rest without motor imagery (n = 3). It was confirmed that there was no EMG activity in the 7 muscles (FDI, ADM, ECR, FCR, Thenar, Flexor digitorum superficialis and Extensor digitorum muscles) under any condition (Fig. 2).

### Experiment 1

After electrodes for EMG recording were attached, subjects sat comfortably in a chair with the right forearm fixed in a horizontal position on an armrest. The hand was kept in a prone position throughout the experiment. The subjects were asked to close their eyes and to keep their muscles relaxed. Before recording the MEPs, all subjects actually tried a power grip of the larger ball several times. Then, the difference between the first person perspective and third person perspective [19] was explained to the subjects, who were subsequently instructed to “relax and imagine the power grip of the larger sized ball for several seconds” while remaining within the first person perspective. After several practice sessions of motor imagery, we gave a verbal reminder to assure that the subjects continued to use the first person perspective, by saying: “Did you use the first person perspective?”. Then, we started the TMS experiments. First, we recorded the MEPs ten times during rest with the “holding posture” but without a ball. Next, the subjects were asked to imagine the “power grip of a larger sized ball” with the right hand utilizing full strength in four different conditions (Fig. 1); (1) PP: “pinching posture” with thumb and index finger without any object, (2) PP+Ball: “pinching posture” with the smaller sized foam ball, (3) HP: “holding posture” with all fingers without any object, (4) HP+Ball: “holding posture” while holding the larger sized foam ball.

To avoid priming effects, a variable interval of 2–4 s elapsed between the verbal command informing subjects to start motor imagery and the TMS. Five consecutive trials of one condition constituted one block. The interval between trials always exceeded 10 s. Four blocks composed of one for each condition were performed in a random order in one session and 4 sessions were conducted with a 5 min rest in between. The total number of trials for each condition was 20. For each condition, MEPs with no background EMG activity were averaged.

### Experiment 2

The experimental procedure was the same as that of experiment 1 except for the type of imagery of an action. The subjects were instructed to relax and imagine the “pincer grip of a smaller sized ball” utilizing the full strength of the right hand. Before the MEPs recording, all subjects actually tried the pincer grip with the smaller ball several times. Before the trial, we recorded the MEPs ten times during rest at the “holding posture” without a ball. The four conditions investigated were the same as in experiment 1.

### Experiment 3

In this experiment, the subjects were instructed to “not to do any motor imagery”. All the other experimental procedures were the same as those of experiment 1 and 2 except for the number of sessions. In the experiment 3, two sessions were employed. Thus, the total number of trials for each condition was 10.

### Experiment 4

Before the MEP recording, all subjects actually tried the power grip several times. In this experiment, the tips of fingers were taped to a wooden board to keep the fingers extended as well as suspended and away from the armrest. The angles of the fingers and wrist were maintained at approximately 180 degrees. Before the trial, we recorded the MEPs ten times during the resting condition. Subjects were asked to imagine the “power grip of the larger sized ball” while utilizing the right hand at full strength, and to keep an extended finger position without any muscle contraction while passively touching a foam hand pad. The hand pad was in contact with the subject's palm and fixed with the same pressure as when holding the ball passively utilizing an elastic band. Four contact conditions were investigated; (1) no contact, (2) lateral half of the palm (ulnar), (3) medial half of the palm (median), (4) the entire palm (entire).

### Experiment 5

The experimental procedure was the same as that of experiment 4 except for the type of imagery of an action. The subjects were instructed to relax and imagine the “pincer grip of a smaller sized ball” utilizing the full strength of the right hand and to keep an extended finger position without any muscle contraction while passively touching a foam hand pad. Before the trial, we recorded the MEPs ten times during the resting condition. The four conditions investigated were the same as in experiment 4.

### Data analysis

Peak-to Peak amplitude of MEPs were measured. In experiments 1, 2, 4 and 5 MEPs were normalized with respect to that of the MEPs obtained in the rest condition for each condition of the four experiments. In experiments 1 ,2 and 3 the differences in the MEPs and the rejection rates of background EMG among the four conditions were tested by a two-way analysis of variance (ANOVA) with repeated measures using the within-subject factors of Posture (pinching and holding), and Object (hold and not-hold). Post hoc analyses were determined utilizing a paired t-test with the Bonferroni correction for dependent samples. In addition, we calculated a ratio of MEP in the hold condition (e.g. PP+Ball) to that in the not-hold condition (e.g. PP) as the “facilitation index” for each posture in experiments 1 and 2. The facilitation index was assessed with paired t-test. In experiments 4 and 5, differences in the MEPs and the rejection rates of background EMG among the conditions were tested utilizing a one-way ANOVA with repeated measures. If the sphericity assumption was violated in Mauchly's sphericity test, the Greenhouse-Geisser correction coefficient epsilon was used to correct the degrees of freedom, and then F and P values were recalculated. Post hoc analyses were determined by utilizing a paired t-test with the Bonferroni correction. All tests were performed with a 95% confidence interval. Data values are expressed as means±one standard error (SE).

## Results

### Experiment 1

The test TMS intensity was 63.0±3.3% of the maximal output of the magnetic stimulator. Data in 9.4% of the total number of trials were excluded from the analysis because of the presence of background EMG activity. The rejection rates were not different across conditions (PP = 10±3%, PP+Ball = 7±3%, HP = 12±5%, HP+Ball = 9±3%).

**Figure 3:**
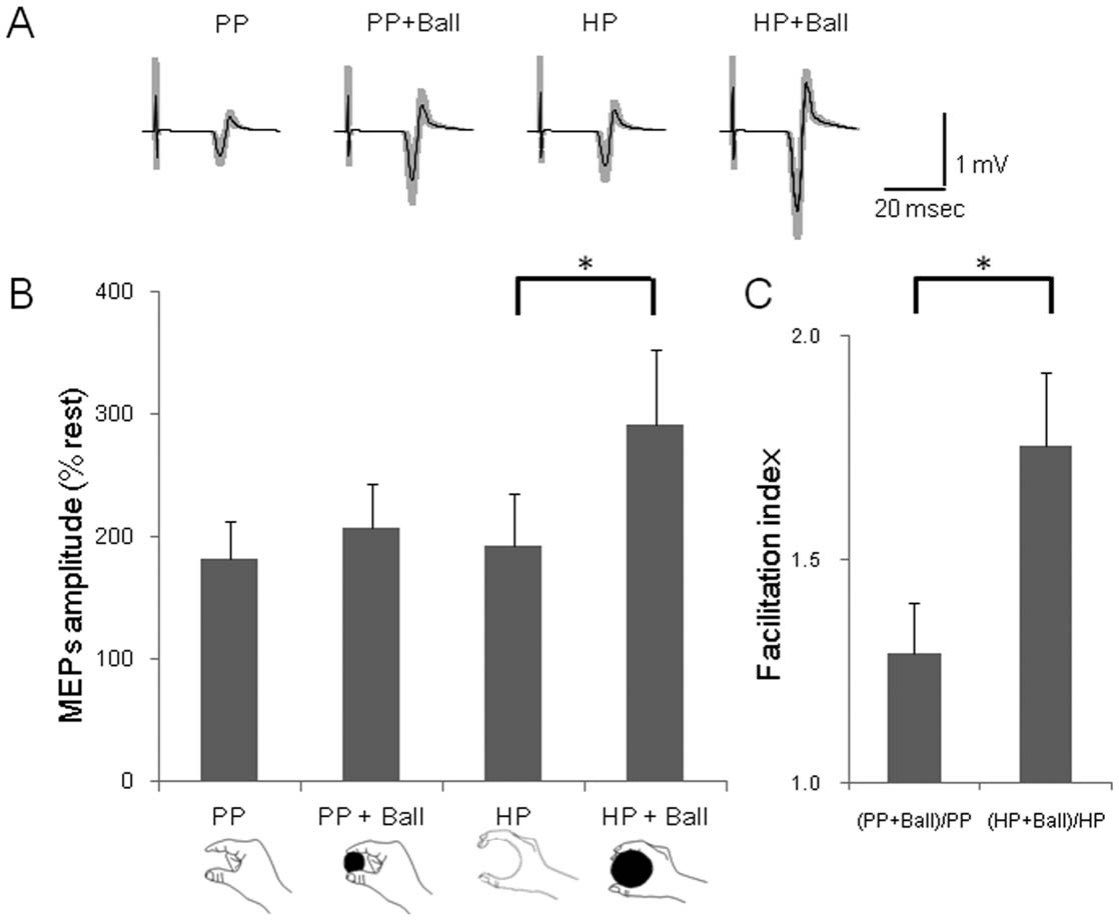
MEPs amplitudes during the holding conditions with imagery of the power grip. (A) An average of 20 trials of MEPs recorded in the FDI during the Pinching Posture (PP), Pinching Posture with a smaller ball (PP+Ball), Holding Posture (HP) and Holding Posture with a larger ball (HP+Ball) conditions. The gray zones indicate ±1 SD. (B) The MEP amplitudes during imagery of the power grip in the FDI under four conditions for 12 subjects. Two-way ANOVA revealed a main effect of object (hold or not-hold) (p<0.05) and an interaction was found between two factors (p<0.05). The MEPs amplitude in the HP+Ball condition was significantly greater than that of the HP condition (p<0.05). (C) The facilitation index in the “holding posture” was significantly greater than that of the “pinching posture” (p<0.05).

The MEPs in the FDI taken from a representative subject are shown in Fig. 3A. The MEP amplitudes in the holding posture while holding the ball condition tended to be larger than those in other conditions. The average amplitudes of the 12 subjects are shown in Fig. 3B. ANOVA for the FDI revealed a main effect of Object [F(1,11) = 8.08, p<0.05]. Furthermore, an interaction was found between two factors [F(1,11) = 6.53, p<0.05]. A paired t-test with the Bonferroni correction showed that the MEP amplitudes of the FDI in the HP+Ball were significantly greater than those of the HP condition (T = 3.53. p<0.05). However, the other three contrasts did not differ significantly (PP vs. PP+Ball, T(11) = 1.17, p>0.05; PP vs. HP, T(11) = 0.42, p>0.05; PP+Ball vs. HP+Ball, T(11) = 2.67, p>0.05). The facilitation index for the “holding posture” was significantly greater than that in the pinching posture (p<0.05) (Fig. 3C). MEPs were also collected from other muscles (ADM, ECR and FCR). However, the MEP amplitudes were quite small. For MEP amplitudes in the ADM, ANOVA showed a main effect of Object [F(1,11) = 9.89, p<0.01] and an interaction effect [F(1,11) = 5.87, p<0.05]. The MEP amplitudes for ADM in the HP+Ball condition were significantly greater than those in the HP condition (p<0.05). For the MEP amplitudes in ECR, ANOVA demonstrated a main effect of Posture [F(1,11) = 6.44, p<0.05]. For the MEP amplitudes in FCR, ANOVA revealed a main effect of Posture [F(1,11) = 52.40, p<0.01].

### Experiment 2

The test TMS intensity was 63.1±3.2% of the maximal output of the magnetic stimulator. Data in 8.4% of the total number of trials were excluded from the analysis because of the presence of background EMG activity. The rejection rates were not different across conditions (PP = 5±2%, PP+Ball = 8±3%, HP = 8±4%, HP+Ball = 14±6%).

**Figure 4:**
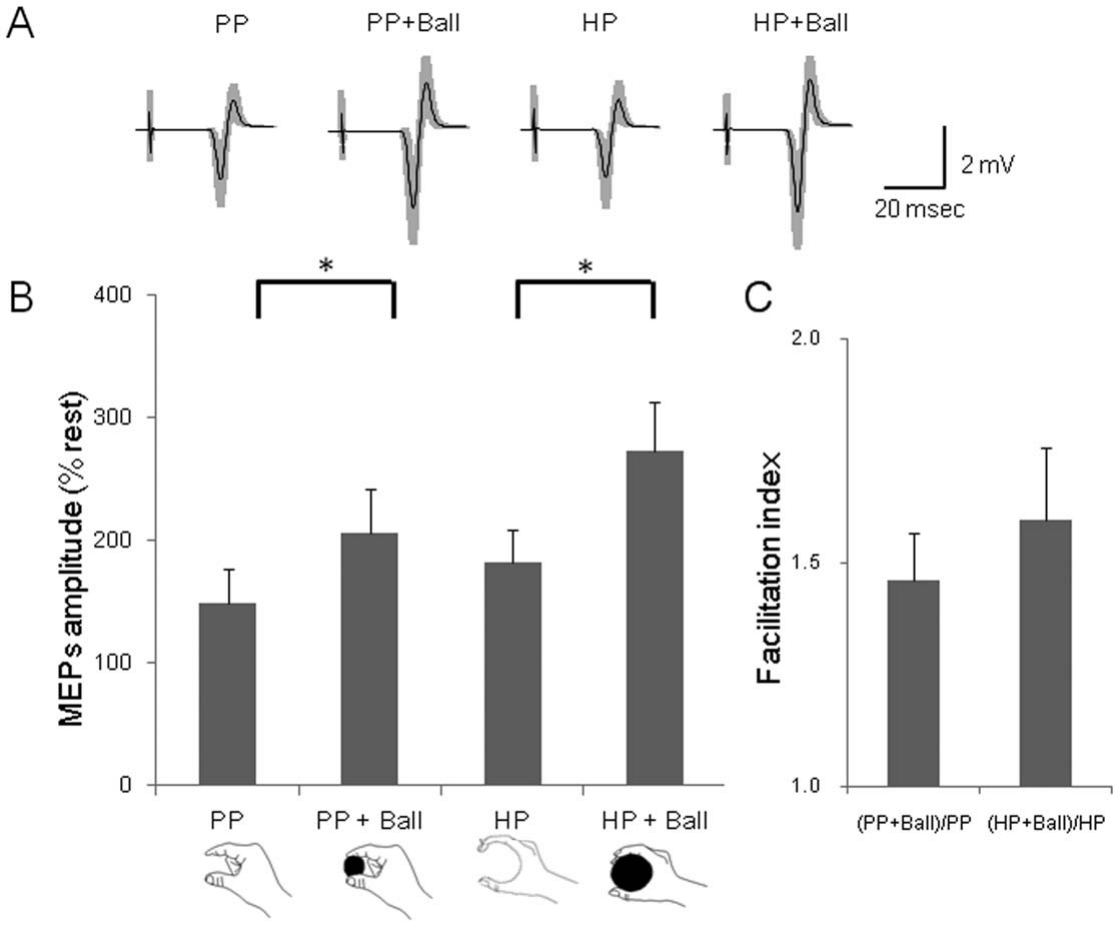
MEPs amplitudes during the holding conditions with imagery of the pincer grip. (A) An average of 20 trials of MEPs recorded in the FDI during the Pinching Posture (PP), Pinching Posture with a smaller ball (PP+Ball), Holding Posture (HP) and Holding Posture with a larger ball (HP+Ball) conditions. The gray zones indicate ±1 SD. (B) The MEP amplitudes during imagery of the pincer grip in the FDI in four conditions for 12 subjects. Two-way ANOVA revealed main effects of object (hold or not-hold) (p<0.05) and Posture (pinching or holding) (p<0.05). The MEP amplitudes in the PP+Ball condition were significantly greater than those of the PP condition (p<0.05) and the HP+Ball condition was significantly greater than that of the HP condition (p<0.05). (C) The facilitation index did not differ between the two postures (p>0.05).

The MEPs in the FDI taken from a representative subject are shown in Fig. 4A. The average amplitudes of the 12 subjects are shown in Fig. 4B. ANOVA for the FDI revealed main effects of Object [F(1,11) = 26.14, p<0.01] and Posture [F(1,11) = 9.45, p<0.05]. A paired t-test with the Bonferroni correction showed that the MEPs amplitudes for FDI in the PP+Ball was significantly greater than those in the PP condition (T(11) = 3.67, p<0.05) and the HP+Ball was significantly greater than those in the HP condition (T(11) = 3.80, p<0.05). However, the contrasts of PP vs. HP (T(11) = 2.90, p>0.05), and PP+Ball vs. HP+Ball (T(11) = 2.37, p>0.05) were not significant. The facilitation index did not differ between the two postures (Fig. 4C). ANOVA for the MEP amplitudes in the ADM showed a main effect of Object [F(1,11) = 7.00, p<0.05]. For the MEP amplitudes in FCR, there was a main effect of Posture [F(1,11) = 9.99, p<0.01]. The MEP amplitudes in ECR did not differ significantly among the four conditions.

### Experiment 3

**Figure 5:**
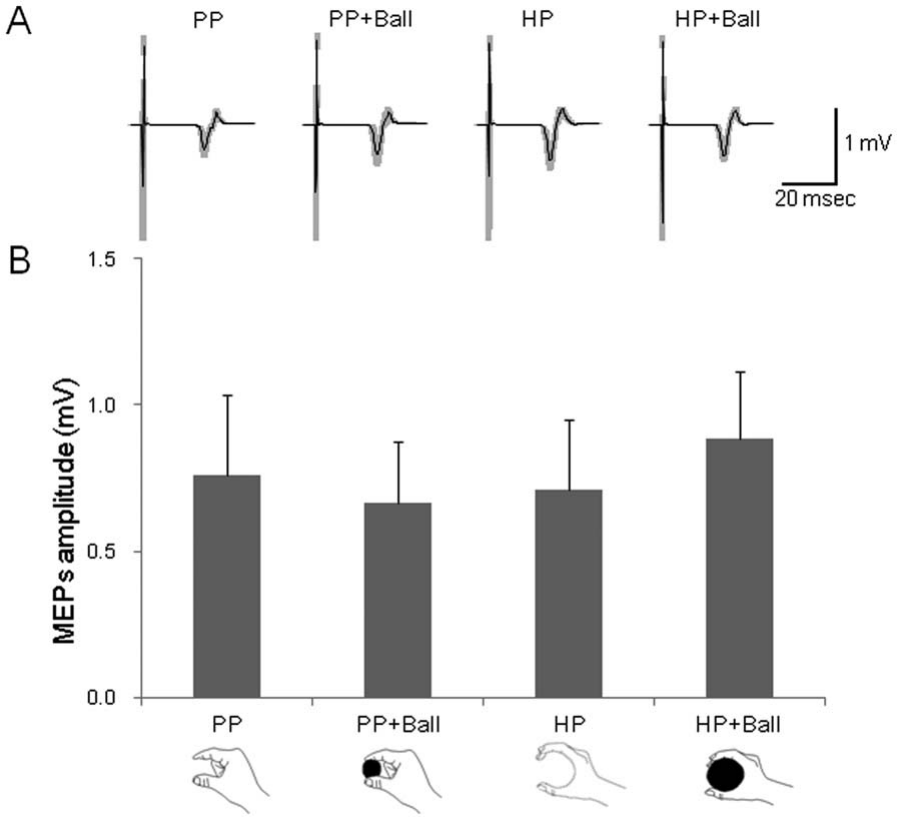
MEPs amplitudes during the holding conditions without motor imagery. (A) An average of 10 trials of MEPs recorded in the FDI during the Pinching Posture (PP), Pinching Posture with a smaller ball (PP+Ball), Holding Posture (HP) and Holding Posture with a larger ball (HP+Ball) conditions. The gray zones indicate ±1 SD. (B) The MEPs amplitudes without motor imagery in the FDI in four conditions for 10 subjects.

The test TMS intensity was 59.9±3.5% of the maximal output of the magnetic stimulator. Data in 2.8% of the total number of trials were excluded from the analysis because of the presence of background EMG activity. The rejection rates were not different across condition (PP = 0±0%, PP+Ball = 4±8%, HP = 0±0%, HP+Ball = 2±3%). The MEPs in the FDI taken from a representative subject are shown in Fig. 5A. The average amplitudes of the 10 subjects are shown in Fig. 5B. The MEP amplitudes in three muscles (FDI, ADM and ECR) did not differ significantly among the four conditions (p>0.05). For the MEP amplitudes in FCR, there was a main effect of Posture [F(1,9) = 8.68, p<0.05]. However, the mean values of MEPs amplitudes in the FCR were smaller than 0.02 mV while those in FDI muscle were greater than 0.6 mV.

### Experiment 4

The test TMS intensity was 64.7±4.9% of the maximal output of the magnetic stimulator. Data in 7.8% of the total number of trials were excluded from the analysis because of the presence of background EMG activity. The rejection rates were not different across condition (no = 6±2%, ulnar = 11± 3%, median = 11±4%, entire = 5±2%).

**Figure 6:**
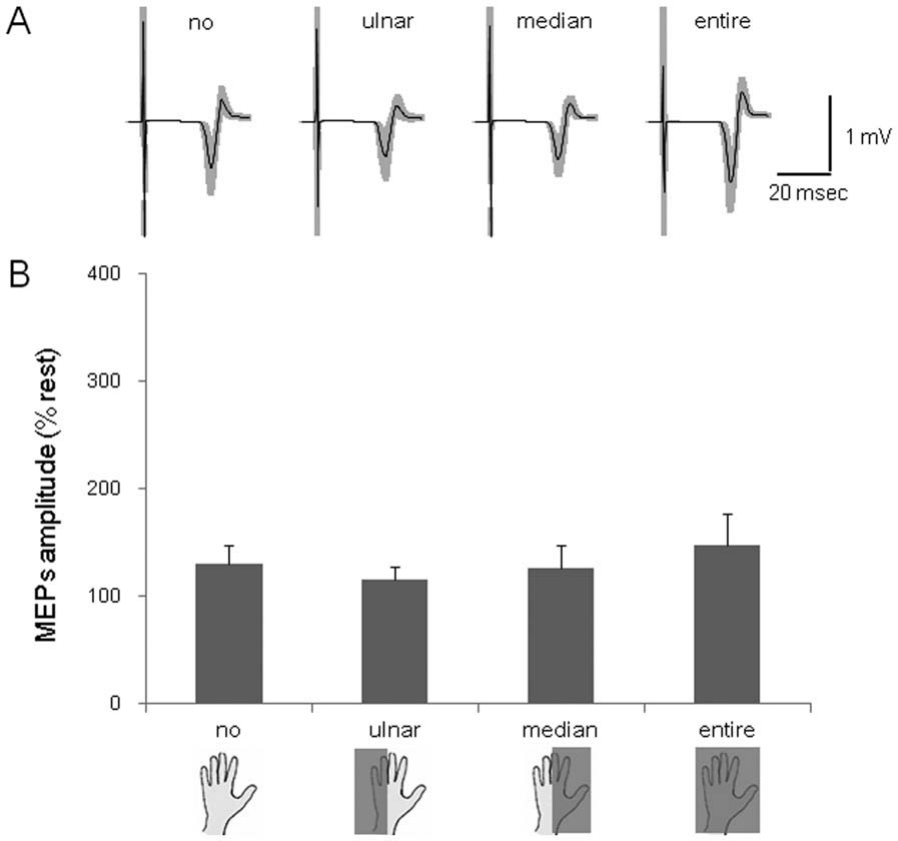
MEPs amplitudes during four conditions with imagery of the power grip. (A) An average of 20 trials of MEPs recorded in the FDI during the no contact, ulnar, medial and entire conditions. The gray zones indicate ±1 SD. (B) The MEP amplitudes during imagery of the power grip in the FDI in four conditions for 12 subjects.

The MEPs in the FDI taken from a representative subject are shown in Fig. 6A. The average amplitudes of the 12 subjects are shown in Fig. 6B. The MEP amplitudes in four muscles (FDI, ADM, ECR and FCR) did not differ significantly in the four conditions (F(3,33) = 1.97, p>0.05; F(1.4,5.5) = 2.11, p>0.05; F(1.78,19.2) = 2.61, p>0.05; F(3,33) = 0.44, p>0.05).

### Experiment 5

**Figure 7:**
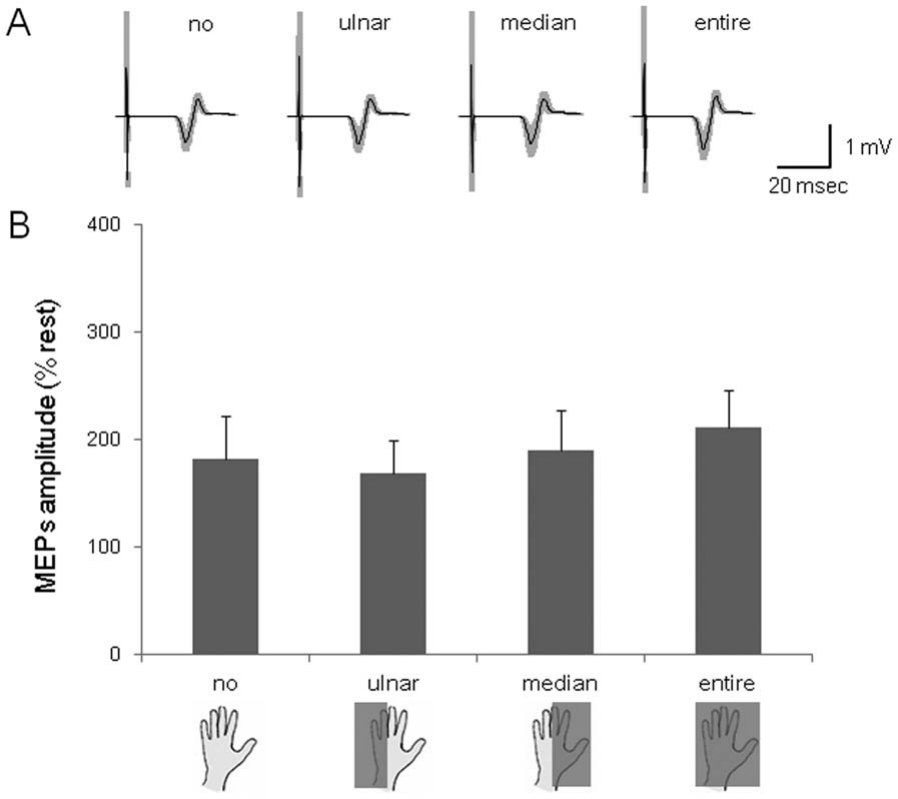
MEPs amplitudes during four conditions with imagery of the pincer grip. (A) An average of 20 trials of MEPs recorded in the FDI during the no contact, ulnar, medial and entire conditions. The gray zones indicate ±1 SD. (B) The MEP amplitudes during imagery of the pincer grip in the FDI in four conditions for 12 subjects.

The test TMS intensity was 58.8±2.6% of the maximal output of the magnetic stimulator. Data in 7.6% of the total number of trials were excluded from the analysis because of the presence of background EMG activity. The rejection rates were not different across condition (no = 7±3%, ulnar = 9±4%, median = 10±4%, entire = 5±3%). The MEPs in the FDI taken from a representative subject are shown in Fig. 7A. The average amplitudes of the 12 subjects are shown in Fig. 7B. The MEP amplitudes in four muscles (FDI, ADM, ECR and FCR) did not differ significantly in the four conditions (F(3,33) = 2.22, p>0.05; F(3,33) = 2.11, p>0.05; F(3,33) = 2.84, p>0.05; F(3,33) = 0.55, p>0.05, respectively).

## Discussion

In our previous study, we showed that during imagery of an action with an object (power grip of a ball), corticospinal excitability was enhanced by holding the object [7]. In the present study, we investigated whether tactile, proprioceptive or both sensory signals are responsible for the enhancement of corticospinal excitability. We monitored the MEPs amplitude during imagery of the power grip (experiment 1) and the pincer grip (experiment 2) during the holding or the pinching posture with or without passively touching a ball. In both experiment 1 and experiment 2 MEPs amplitudes during motor imagery were increased by touching balls while the posture was same as the imagined action. The amplitudes of MEPs in the ADM showed the same tendency as the FDI. The MEPs amplitudes in FCR or ECR also showed the effect of the posture. However, the MEP amplitudes were small and had a large variation in muscles other than the FDI, probably because the site of the TMS coil was not optimal for the muscles. To clarify the influence of posture or touching the object in these muscles, future studies will need to be performed.

The enhancement of corticospinal excitability by touching a ball could not have been caused by unintentional contraction of the muscles responsible for the actions, because the preliminary experiment showed that the hand and arm muscles were completely inactive during the four conditions investigated (Fig. 2). In addition, enhancement of corticospinal excitability is not merely the effect of the two different posture conditions or to just holding a ball, because no MEPs modulation occurred in the same condition as the experiment 1 and 2 but without motor imagery (experiment 3). This result was consistent with what we found in our previous study [7], in that performing “motor imagery” is a prerequisite for the enhancement of corticospinal excitability. In both experiment 1 and experiment 2, corticospinal excitability was enhanced during the tasks of passively holding/pinching a ball as compared with those without a ball, although the actual postures were the same in both conditions. This means that tactile signals generated by ball touching play an important role in the enhancement of corticospinal excitability. Then, is the tactile signal alone responsible for the enhancement of corticospinal excitability during motor imagery? To test this question, in experiments 4 and 5 we examined the effect of touching various areas of an open palm on corticospinal excitability during imagery of the “power grip” and “pincer grip”. However, the MEP amplitudes were not modulated by touching either half of the palm (ulnar or median) or the entire palm (Fig. 6B, 7B). Especially, in the case of imagery of a pincer grip of the smaller ball, even touching larger areas of the entire palm than would occur in the real action did not elicit enhancement of corticospinal excitability. Thus, the enhancement of corticospinal excitability during motor imagery was not produced merely by the tactile input.

As for hand actions without objects, corticospinal excitability during motor imagery is greater when the actual and the imagined hand posture are congruent than when the two are incongruent [9], [12]. This suggested that propioceptive input from the hand generated with a congruent posture affects the enhancement of corticospinal excitability during motor imagery. However, in the present study, there was no significant modulation of MEPs when postures alone are altered (PP vs. HP). Therefore, for modulation of corticospinal excitability to occur during motor imagery of actions “with object”, a combination of proprioceptive and tactile inputs is needed.

During imagery of the power grip of the larger sized ball, MEPs amplitude increased only during the passive holding of the larger sized ball (congruent) (experiment 1). In contrast, during imagery of the pincer grip of the smaller sized ball, MEPs amplitude increased not only during pinching the smaller sized ball (congruent) but also during holding the larger sized ball (incongruent) (experiment 2). This discrepancy might be explained by the “action capability” of the subject's posture. From the holding posture both the power and the pincer grip can be performed smoothly. On the other hand, from the pinching posture only the pincer grip is able to be initiated. To perform the power grip the thumb and fingers would need to be initially opened. Thus, it might be that for the enhancement of corticospinal excitability during motor imagery, the hand should be at least in a posture from which the imagined action could be performed smoothly.

The visual presence of objects modulates the reaction time of actions with the object, the activity of motor related region, and corticospinal excitability [20]–[22]. Ellis and Tucker (2000) demonstrated that visual presentation of an object, which is grasped with a precision grip, reduces the reaction time for a precision grip (congruent) and not for a power grip (incongruent), and vice versa [20]. The effects of visual stimuli on actions appear with the stimulus-response compatibility of a particular action [20], [21], [23]. The present study, done in subjects with eyes closed, suggests the possibility that the influence of a stimulus-response compatibility exists also for somatosensory stimuli in the case of actions with objects. However, on what aspects of action the compatibility depends, actual posture (grasp type) and/or objects size should be elucidated in the future studies.

In the present study we asked subjects to use the kinesthetic motor imagery (first person perspective) which has been distinguished from the visual motor imagery (third person perspective) [9], [19]. Fourkas et al. (2006) report that corticospinal excitability during kinesthetic motor imagery of a thumb-palm opposition movement is greater when the actual posture and imagined posture are congruent than when they are incongruent, and the congruency of actual and imagined postures does not influence visual motor imagery [9]. In the present study, the effects of somatosensory inputs on corticospinal excitability were investigated only during kinesthetic motor imagery. Effects that occur during visual motor imagery might be different from those observed in the present study.

Fourkas et al. (2008) report that the vividness of motor imagery is correlated with corticospinal excitability during motor imagery [24]. Subjects with the ability to create a more vivid experience of motor imagery, as measured by a self-report questionnaire [25], can improve motor skills by mental practice to a greater degree than can those with a low ability [26]. If motor imagery of a tool using action were done while holding the same object, that should improve motor skills even more effectively. Thus, mental practice of an object using action while touching the object being imaged should be of benefit for both patients and athletes.
